# Evaluating CO_2_ Capture in Aqueous Amine Solution by Using a New Microbubble System

**DOI:** 10.1021/acsomega.6c01346

**Published:** 2026-05-29

**Authors:** Rafael Garcia Candido, Thiago Galeote Tabuti, Leandro Gonçalves de Aguiar, Pedro Felipe Arce, João Paulo Alves Silva, Lívia Melo Carneiro, Koiti Araki, Eduardo Rezende Triboni

**Affiliations:** † Engineering School of Lorena, Chemistry Department, São Paulo University, Lorena, São Paulo 12602-810, Brazil; ‡ Chemistry Institute, São Paulo University, São Paulo, São Paulo 05508-000, Brazil

## Abstract

The present study evaluated two systems, one of the CO_2_ jets and another of microbubbles, for CO_2_ capture by aqueous amine solutions, comparing the performance of monoethanolamine (MEA) and triethanolamine (tertiary amine (TEA)). The capture efficiency was calculated through the mass variation measurements. The results revealed that the CO_2_ jet system did not achieve satisfactory results. However, using the microbubble system, MEA exhibited the highest efficiency, surpassing the stoichiometric value (0.5 mol of CO_2_/mol of amine), and TEA was close to its theoretical limit. The presence of water was able to enhance CO_2_ absorption in this system with an optimal amine-to-water ratio identified for both solvents. In addition, mathematical modeling was carried out to determine the reaction kinetics and mass transfer constant for the microbubble system and demonstrated strong agreement with experimental data. Conductor-like screening model for real solvents was applied to predict thermodynamic properties and validate the results found in the other analysis. The new microbubble system considerably improved CO_2_ capture efficiency, decreased reaction time, and enhanced mass transfer, offering a promising alternative for large-scale carbon capture applications.

## Graphical abstract

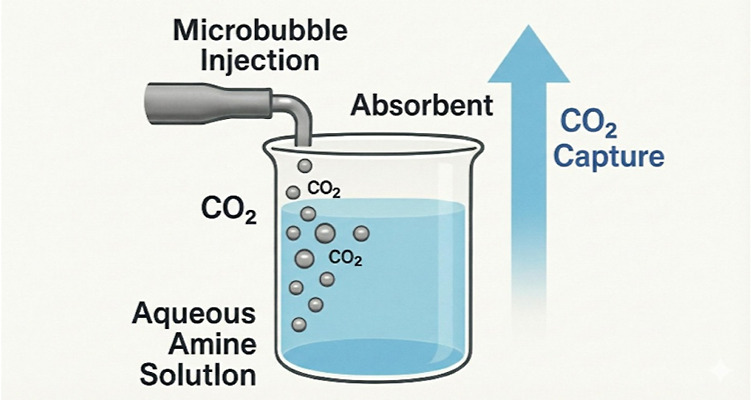

## 1 Introduction

Since the Industrial Revolution, CO_2_ emissions have surged, reaching over 426–428 ppm in 2025, compared to around 300 ppm in the 1950s.^1,2^ This increase disrupts the carbon cycle and drives climate change. Beyond vehicles, industries such as cement, steel, chemical, and fossil fuel power plants contribute significantly.^3^ As a result, CO_2_ separation technologies are expanding, with growing research on capture, storage, and reuse to mitigate global warming and energy demands.^4,5^

Absorption is the most extensively studied and widely applied technique for the capture of CO_2_. It entails the dissolution of CO_2_ into a solvent, which may either undergo a chemical reaction with the absorbent (chemical solvents).^6^ Liquid absorbents based on amine compounds have been employed in industrial CO_2_ capture processes via absorption since the 1930s. The widespread industrial application of amine-based absorbents can be attributed to their low cost and high CO_2_ absorption capacity, distinguishing them from those of alternative absorbents. Furthermore, such a process operates under relatively mild conditions, further contributing to its industrial viability.^7,8^

Various amine species have been employed in CO_2_ capture processes, with alkanolamines being the most utilized. To date, monoethanolamine (MEA)-based solvents are the most suitable for chemical absorption due to its high CO_2_ absorption rate and relatively low cost. Consequently, several carbon capture and storage (CCS) facilities using MEA have been successfully implemented on a commercial scale in the United States, Canada, and China.^9,10^ MEA has also been explored in emerging CO_2_ removal applications, such as waste-to-gas process.^11^ The CO_2_ capture takes place by a zwitterionic mechanism in which a zwitterion intermediate is first formed (eq 1), then to react with another MEA molecule (eq 2), leading to the generation of protonated amines and carbamate ions (eq 3)^12^

1 $$Amine+{CO}_{2}⇌{AmineH}^{+}{COO}^{-}$$

2 $$Amine+{Amine}^{+}{COO}^{-}⇌{AmineH}^{+}+{AmineCOO}^{-}$$

3 $$2Amine+{CO}_{2}⇌{AmineH}^{+}+{AmineCOO}^{-}$$

where Amine, AmineCOO^–^, and AmineH^+^ represent free amines, carbamate ions, and protonated amines, respectively.

However, MEA has two inherent drawbacks, which are its low CO_2_ absorption capacity and moderate reaction kinetics requiring larger absorption columns. Such disadvantages increase capital costs compared to more rapidly reacting amines.^13,14^

Tertiary amines (TAs), as triethanolamine (tertiary amine (TEA)), have been an alternative to address certain limitations of MEA-based solvents because of their higher theoretical efficiency that is a 1 to 1 reaction stoichiometry of CO_2_ per amine, in spite of possessing a lower CO_2_ absorption rate compared to MEA.^15^ In addition, solvent degradation and losses are reduced due to their thermal stability and low volatility.^16,17^ Another important feature is that the reaction with CO_2_ provides bicarbonate ions (HCO3-) instead of carbamate ions, which facilitates the release of CO_2_ and the recovery of TA.^15,18^ TA reacts with CO_2_ through a base-catalyzed hydration mechanism by which it acts as a catalyst and proton acceptor for the CO_2_ and H_2_O reactions, resulting in the formation of bicarbonate ions^19^

4 $$AmineT+H_{2}O+{CO}_{2}⇌{AmineTH}^{+}+{HCO}_{3}^{-}$$

where AmineT and AmineTH^+^ represent the TA (TEA) and the protonated form of the TA (TEA), respectively.

The influence of water has been evaluated on the performance of aqueous amine absorbents, although the underlying mechanisms are not well understood. As a general assumption, water is expected to affect the absorption process by both taking part in the chemical reactions and modifying the solvation environment.^20^ Therefore, it is essential to determine the optimal water content in the system, taking into account its influence on solvent viscosity, CO_2_ solubility, and the heat of absorption.

Microbubble technology has been widely investigated across various gas–liquid processes owing to its improved mass transfer performance.^21^ For instance, microbubbles have been applied in water and wastewater treatment for improved aeration and pollutant removal,^22^ in flotation and mineral processing to enhance separation efficiency,^23^ and in gas–liquid reaction engineering to boost uptake and reaction rates of dissolved gases.^24^ These applications leverage the high interfacial area and prolonged residence times of microbubbles, which significantly increase mass transfer compared to conventional bubbling.^25^ Recently, researchers have established that microbubble technology offers substantial advantages in the implementation of CCS. These benefits encompass enhanced gas separation, the efficient capture of CO_2_ using microbubbles, its storage in saline aquifers or oil reservoirs, and the facilitation of microbubble-based phase transitions in CO_2_ hydrate formation.^26–28^ Moreover, in opposition to large bubbles (∼1 mm), microbubbles (tens of micrometers) have a higher surface area, leading to lower surface tension, shrinkage, and rapid dissolution.^29,30^ Their low-rise velocity extends residence time, preventing coalescence and enhancing gas–liquid contact, which increases dissolved gas concentration.^31^ From an experimental standpoint, the performance of microbubble-based gas–liquid systems has typically been evaluated using a combination of gravimetric measurements, gas uptake curves, dissolved gas concentration monitoring, and residence-time analysis, depending on the scale and objective of the study. These approaches allow indirect yet reliable assessment of mass transfer enhancement without the need for complex hydrodynamic diagnostics.^25^

Process modeling techniques are essential for analyzing chemical and industrial processes and guiding the design of innovative solutions.^32,33^ While CO_2_–amine reaction kinetics have been studied for decades, the exact mechanism in aqueous systems is not fully clear. Research continues to uncover these mechanisms and their impact on absorption efficiency.^34,35^

The conductor-like screening model for real solvents (COSMO-RS) model is an efficient and robust predictive tool that can estimate key thermodynamic properties—such as activity coefficients, solubility, Gibbs free energy, and Henry’s law constant—based on quantum chemical calculations and statistical thermodynamics. It uses the three-dimensional distribution of screening charge densities (σ) on molecular surfaces, represented by σ-profiles, to qualitatively describe molecular interactions. Applicable to solvents like aqueous amines, COSMO-RS enables the evaluation of solvent suitability for gas capture through analysis of σ- profiles, σ-potentials, and interaction energies with gaseous analytes.^36,37^

Although microbubble technology has been explored in various gas–liquid processes, its application to CO_2_ capture via chemically reactive aqueous amine systems is not largely explored to date. Herein, a microbubble-based CO_2_ capture system operating at ambient pressure was developed, achieving high capture efficiencies within significantly reduced reaction times. In addition, two different systems CO_2_-jet and microbubble injection, were compared toward their CO_2_-capture efficiency in aqueous amine MEA and TEA solutions. Moreover, we determined the reaction kinetic values for the microbubble injection system by process modeling and predicted the thermodynamic properties of the amines using the COSMO-RS model. The CO_2_-microbubble injection approach offers a promising pathway to reduce energy consumption while increasing productivity in carbon capture processes.

## 2 Methodology

### 2.1 Materials

MEA (CAS no. 141-43-5) was purchased from Aldrich, and TEA (CAS No. 102-71-6) was purchased from Synth. Amines were reagent-grade (purity higher than 99%) and were used as received without further purification. No additional solvents were used in this study. The CO_2_ used in the capture tests presented a purity grade of 100%.

### 2.2 CO_2_ Capture Processes

#### 2.2.1 System 1

System 1 for CO_2_ capture was carried out employing a CO_2_ jet that was directly injected by a needle into the amine solutions (Figure 1) at a controlled flow rate of 6 L/min. At specified time intervals, the mass variation within the system was measured and utilized to calculate the capture efficiency (mol of CO_2_ captured/mol of amine). Mass measurements were carried out using an analytical balance with a readability of 0.001 g, and the associated standard error was on the order of balance resolution. All reactions were carried out under ambient-temperature and -pressure conditions. Each experiment was performed in duplicate to ensure the reproducibility. Table 1 presents the different dilutions tested. The solutions were prepared by using amine and distilled water.

**Figure 1:**
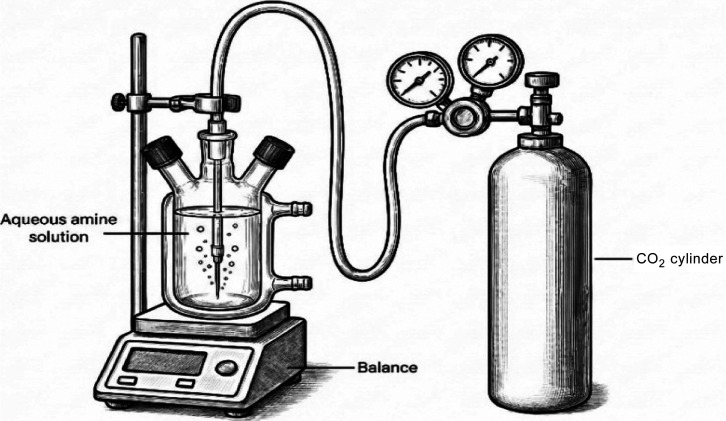
System for CO_2_ capture using CO_2_ jet.

**Table 1:** Process Conditions and Corresponding Labels^a^

amine/water volume ratio	volume (mL)	MEA	TEA
1:0	25	MEA1	TEA1
1:1	24	MEA2	TEA2
1:2	24	MEA3	TEA3

a MEA1, MEA2, and MEA3 (and analogously TEA1, TEA2, and TEA3) correspond to different amine–water compositions, as specified in the table.

#### 2.2.2 System 2

System 2 for CO_2_ capture was conducted by using the system illustrated in Figure 2.

**Figure 2:**
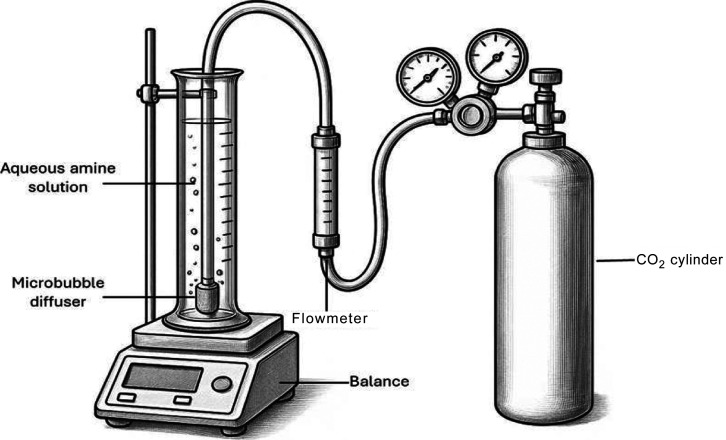
System for CO_2_ capture using microbubbles.

CO_2_ microbubbles were directly injected into the amine solutions (Table 2), using a stainless-steel diffuser with a porous size of 2 μm, at a controlled flow rate of 0.5 L/min. At specified time intervals, the mass variation within the system was measured and utilized to calculate the capture efficiency (mol of CO_2_ captured/mol amine). Mass measurements were carried out using an analytical balance with a readability of 0.001 g, and the associated standard error was on the order of the balance resolution. All experiments were conducted under ambient conditions in an open system. Temperature was monitored throughout the experiments and remained nearly constant, with variations not exceeding 5 °C. Because the experiments were performed under atmospheric pressure in an open reactor, pressure variations were not monitored and were assumed constant.

**Table 2:** Process Conditions and Corresponding Labels

amine/water volume ratio	volume (mL)	MEA	TEA
1:0	25	M1	T1
1:0.5	24	M2	T2
1:1	24	M3	T3
1:2	24	M4	T4
1:4	25	M5	T5
1:5	24	M6	T6

Each experiment was performed in duplicate to ensure reproducibility. Table 2 shows the different dilutions that were tested. The solutions were prepared by using amine and distilled water.

### 2.3 Infrared Spectroscopy

Fourier transform IR (FTIR) spectroscopy analyses were conducted using a Thermo Nicolet iS10 FTIR spectrometer (Thermo Scientific, Waltham, MA, USA) equipped with an attenuated total reflectance accessory. Spectra were acquired within the wavenumber range of 4000–600 cm^–1^, utilizing 32 scans with a spectral resolution of 4 cm^–1^.

### 2.4 Modeling

#### 2.4.1 Reaction-Transport Model

The CO_2_ capture processes using MEA and TEA were mathematically described in terms of reaction kinetics and mass transfer phenomena. Various mechanisms have been reported in the literature for each capture process, and distinct experimental behaviors have also been observed. Therefore, each system is described separately in the following subsections.

#### 2.4.2 Modeling CO_2_ Capture by MEA Solution Assumptions

- There is a period of approximately 100 s for the complete filling of the medium.
- With CO_2_ bubbles, leading to a quasi-steady interfacial conditions (Figure 3). Experimental measurements showed that during the first ∼100 s, no measurable mass increase was detected, indicating that effective CO_2_ absorption had not yet started. This period was therefore considered a transient stabilization phase prior to model application.
- After 100 s, the total bubble area remains practically constant until the end of the process (the gas phase present in the medium acts as a CO_2_ reservoir maintained under the same conditions throughout the process).
- Bubbles are evenly distributed throughout the solution. The assumption of a constant bubble area was supported by visual observation, which indicated stable and uniform microbubble generation from the diffuser throughout the experimental runs. According to the manufacturer’s specifications, the diffuser pore size limits the maximum bubble diameter to approximately 2 μm, defining the range of bubble sizes for which the model assumptions are valid.
- Pseudosteady state for CO_2_ flow.
- Isothermal processes.

**Figure 3:**
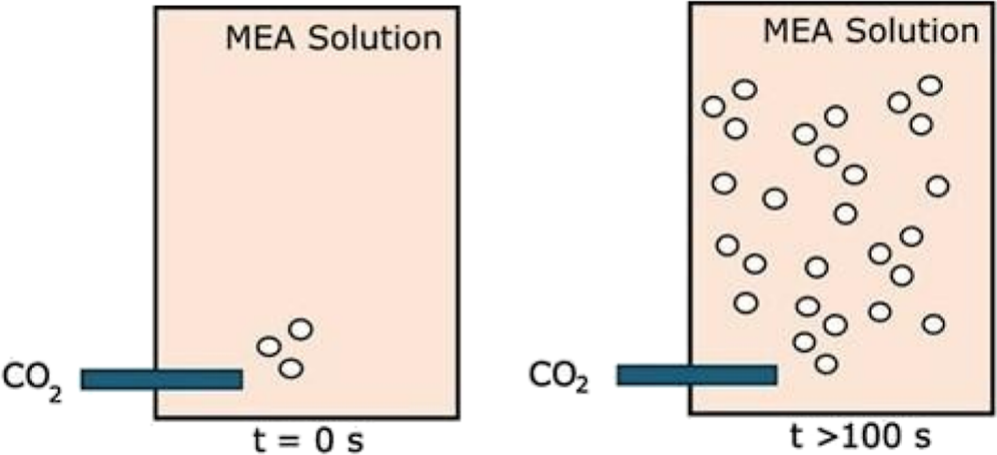
Process stabilization period.

The reactions considered in the process of CO_2_ capture by MEA solution and the respective references are listed as follows^15,35^

5 $$CO_{2}2MEA\begin{array}{l} k_{1f}\\ ⇄\\ k_{1r} \end{array}MEACOO^{-}MEAH^{+}$$

6 $$CO_{2}+H_{2}O\begin{array}{l} k_{2f}\\ ⇄\\ k_{2r} \end{array}H_{2}CO_{3}$$

As illustrated in Figure 2, after 100 s of stabilization, the system was considered to have reached a pseudosteady state, with a constant bubble concentration. Therefore, a molar balance can be performed for each species exclusively in the liquid phase, as follows

7 $$\frac{d\left[MEA\right]}{dt}=-2\left(k_{1f}\left[CO_{2}\right]{\left[MEA\right]}^{2}-k_{1r}\left[MEACOO^{-}\right]\left[MEAH^{+}\right]\right)$$

8 $$\frac{d\left[CO_{2}\right]}{dt}=\frac{k_{AM}A\left({\left[CO_{2}\right]}_{G}-\left[CO_{2}\right]\right)}{V}-\left(k_{1f}\left[CO_{2}\right]{\left[MEA\right]}^{2}-k_{1r}\left[MEACOO^{-}\right]\left[MEAH^{+}\right]\right)-\left(k_{2f}\left[CO_{2}\right]\left[H_{2}O\right]-k_{2r}\left[H_{2}CO_{3}\right]\right)$$

By applying the assumption of a constant bubble area (*A*), a mass transfer constant of CO_2_ in the MEA solution (kTM) can be determined

9 $$k_{TM}=k_{AM}A$$

where *k*_AM_ is the mass transfer coefficient of CO_2_ in the MEA solution in L cm^–2^ min^–1^.

By substituting (9) into (8) and applying the assumption of a pseudosteady state for CO_2_, meaning that all CO_2_ transferred to the liquid phase is immediately consumed, we obtain

10 $$\frac{d\left[CO_{2}\right]}{dt}=0$$

11 $$\left[CO_{2}\right]=\frac{k_{1r}\left[MEACOO^{-}\right]\left[MEAH^{+}\right]+k_{2r}\left[H_{2}CO_{3}\right]+\frac{k_{TM}}{V}{\left[CO_{2}\right]}_{G}}{\frac{k_{TM}}{V}+k_{1f}{\left[MEA\right]}^{2}+k_{2f}\left[H_{2}O\right]}$$

12 $$\frac{d\left[MEACOO^{-}\right]}{dt}=\left(k_{1f}\left[CO_{2}\right]{\left[MEA\right]}^{2}-k_{1r}\left[MEACOO^{-}\right]\left[MEAH^{+}\right]\right)$$

13 $$\frac{d\left[MEAH^{+}\right]}{dt}=\left(k_{1f}\left[CO_{2}\right]{\left[MEA\right]}^{2}-k_{1r}\left[MEACOO^{-}\right]\left[MEAH^{+}\right]\right)$$

14 $$\frac{d\left[H_{2}O\right]}{dt}=-\left(k_{2f}\left[CO_{2}\right]\left[H_{2}O\right]-k_{2r}\left[H_{2}CO_{3}\right]\right)$$

15 $$\frac{d\left[H_{2}CO_{3}\right]}{dt}=\left(k_{2f}\left[CO_{2}\right]\left[H_{2}O\right]-k_{2r}\left[H_{2}CO_{3}\right]\right)$$

16 $$\frac{d{\left[CO_{2}\right]}_{C}}{dt}=\left(k_{1f}\left[CO_{2}\right]{\left[MEA\right]}^{2}-k_{1r}\left[MEACOO^{-}\right]\left[MEAH^{+}\right]\right)+\left(k_{2f}\left[CO_{2}\right]\left[H_{2}O\right]-k_{2r}\left[H_{2}CO_{3}\right]\right)$$

where the terms in brackets represent molar concentrations of species in the liquid phase. [CO_2_] is the molar concentration of captured CO_2,_ and [CO_2_]*G* is the concentration of CO_2_ in the gas phase.

The system of algebraic-differential eqs 10–16 was solved by numerical integration using the ODE algorithm in Scilab.

#### 2.4.3 Modeling CO_2_ Capture by TEA Solution Assumptions

- The total bubble area remains practically constant until the end of the process (the gas–liquid interface).
- Phase present in the medium acts as a CO_2_ reservoir maintained under the same conditions throughout the process.
- Bubbles are evenly distributed throughout the solution. The assumption of a constant bubble area was supported by visual observation, which indicated stable and uniform microbubble generation from the diffuser throughout the experimental runs. According to the manufacturer’s specifications, the diffuser pore size limits the maximum bubble diameter to approximately 2 μm, defining the range of bubble sizes for which the model assumptions are valid.
- Pseudosteady state for the CO_2_ flow.
- Isothermal processes.

The reactions considered in the process of CO_2_ capture by TEA solution and the respective reference are listed as follows^15^

17 $$TEA+H_{2}O\begin{array}{l} k_{3f}\\ ⇄\\ k_{3r} \end{array}TEAH^{+}+OH^{-}$$

18 $$CO_{2}+OH^{-}\begin{array}{l} k_{4f}\\ ⇄\\ k_{4r} \end{array}HCO_{3}^{-}$$

Differently from MEA and probably due to a lower superficial tension, in the CO_2_ capture process using TEA, a constant bubble concentration is maintained from time zero. Therefore, a molar balance can be performed for each species exclusively in the liquid phase, as follows

19 $$\frac{d\left[CO_{2}\right]}{dt}=\frac{k_{TT}\left({\left[CO_{2}\right]}_{s}-\left[CO_{2}\right]\right)}{V}-k_{4f}\left[CO_{2}\right]\left[OH^{-}\right]+k_{4r}\left[HCO_{3}^{-}\right]$$

20 $$\frac{d\left[TEA\right]}{dt}=-k_{3f}\left[TEA\right]\left[H_{2}O\right]+k_{3r}\left[TEAH^{+}\right]\left[OH^{-}\right]$$

21 $$\frac{d\left[H_{2}O\right]}{dt}=-k_{3f}\left[TEA\right]\left[H_{2}O\right]+k_{3r}\left[TEAH^{+}\right]\left[OH^{-}\right]$$

22 $$\frac{d\left[HCO_{3}^{-}\right]}{dt}=k_{4f}\left[CO_{2}\right]\left[OH^{-}\right]-k_{4r}\left[HCO_{3}^{-}\right]$$

23 $$\frac{d\left[OH^{-}\right]}{dt}=k_{3f}\left[TEA\right]\left[H_{2}O\right]-k_{3r}\left[TEAH^{+}\right]\left[OH^{-}\right]-k_{4f}\left[CO_{2}\right]\left[OH^{-}\right]+k_{4r}\left[HCO_{3}^{-}\right]$$

24 $$\frac{d\left[TEAH^{+}\right]}{dt}=k_{3f}\left[TEA\right]\left[H_{2}O\right]-k_{3r}\left[TEAH^{+}\right]\left[OH^{-}\right]$$

25 $$\frac{d{\left[CO_{2}\right]}_{c}}{dt}=\frac{k_{TT}\left({\left[CO_{2}\right]}_{s}-\left[CO_{2}\right]\right)}{V}$$

The system of algebraic-differential eqs 19–25 was solved by numerical integration using the ODE algorithm in Scilab.

### 2.5 Thermodynamic Properties

In this study, COSMO-RS approaches were employed to predict the thermodynamic properties of the hybrid solvent components (i.e., aqueous amine) in relation to CO_2_, as well as to evaluate their interaction energy with CO_2_ molecules. Although alternative thermodynamic models based on electrolyte or activity-coefficient frameworks have been successfully applied to specific amine–CO_2_ systems, they typically require extensive experimental parametrization. In contrast, COSMO-RS was selected in this work for its a priori predictive capability and its suitability for qualitative comparison of solvent–CO_2_ interactions under the conditions investigated.^37^

Prior to the predictions, the optimized structures of the hybrid solvent components were obtained from the COSMOthermX database. Density functional theory calculations were carried out using the def-TZVP basis set and the B3-LYP functional. Predictions of all relevant thermodynamic properties were performed using COSMOthermX (version 19.0.4) with the BP-TZVP parametrization. In the COSMO-RS prediction process, solute molecules are initially embedded in a virtual conductor constructed through the continuum solvation model. In this model, the solvent is treated as a continuous medium with a uniform dielectric constant rather than as discrete molecules.

The virtual conductor acts as an idealized environment in which the solute is placed in a hypothetical conductive medium. The shielding charge density distribution (σ-profile) is then calculated for the solute using quantum chemical methods. The σ-profile describes how the virtual conductor screens the electric field of the solute, resulting in a specific charge distribution on the solute’s surface. This three-dimensional surface charge density distribution can be transformed into a histogram-type plot known as the σ-profile, *P*(σ), as expressed by the following equation

26 $$P\left(\sigma \right)=\sum_{i}§iP^{§i}$$

where §*i* denotes the mole fraction of segment *i*, *P* ^§*i*^ refers to the σ-profile of segment *i*, and *i* represents a specific component in the mixture. The σ-profile describes the polarity of the molecules, while the electrostatic potential derived from the σ-profile, known as the σ-potential, characterizes the interaction behavior and the affinity between molecules within the system. The σ-potential, μ­(σ), of the molecules is calculated using eq 27, based on the σ-profile obtained from Klamt’s equation

27 $$\mu \backslash \backslash \backslash \left(\sigma \right)=-RT\ln(\int d\sigma^{\prime }P\left(\sigma^{\prime }\right)\exp\left[-\frac{1}{2}\alpha^{\prime }{\left(\sigma +\sigma^{\prime }\right)}^{2}-\frac{\mu^{\prime }\left(\sigma^{\prime }\right)}{RT}\right]$$

where α′ refers to a general interaction fitting parameter used to describe the energy of the geometrically optimized molecular structure, and μ­(σ) denotes the chemical potential.

In addition to estimating essential thermodynamic properties, COSMO-RS can also be employed to predict the interaction energy within the studied system, specifically between aqueous amines (ethanolamines and triethanolamines). Based on the σ-profile and σ-potential, various interaction energies and hydrogen-bonding properties can be predicted, including misfit interaction energy (*E*_MF_), van der Waals interaction energy (*E*_vdW_), HB acceptor moment (MHBA), and hydrogen-bonding energy (*E*_HB_). The total energy (*E*_total_) is the sum of *E*_HB_, *E*_vdW_, and *E*_MF_. Mathematically, the energies are described using the equation

28 $$E_{MF}\left(\sigma ,\sigma^{\prime }\right)=\alpha_{eff}\left(\frac{\alpha^{\prime }}{2}\right)\left(\sigma +\sigma^{\prime }\right)2$$

29 EHB=αeffCHBmin(0,σσ′+σHB)2

30 $$E_{vdW}=\alpha_{eff}\left(\tau_{v}d^{w}+\tau_{v}d^{w^{\prime }}\right)$$

where α_eff_ represents the effective contact area between two surface segments, c_HB_ denotes the HB interaction strength coefficient, σ_HB_ is the charge density threshold for hydrogen bonding, while the general fitting parameter for van der Waals interactions is represented by τ_v_d^w^ + τ_v_d^w^′.

## 3 Results and Discussion

### 3.1 CO_2_ Capture Efficiency

Figure 4 exhibits the shows of CO_2_ capture for MEA and TEA in different amine concentrations for system 1. For both systems, the efficiency correlation (mol CO_2_/mol amine) is directly related to how efficiently the available reactive sites of amines were utilized by the reaction system. This value does not directly represent the amount of captured CO_2_.

**Figure 4:**
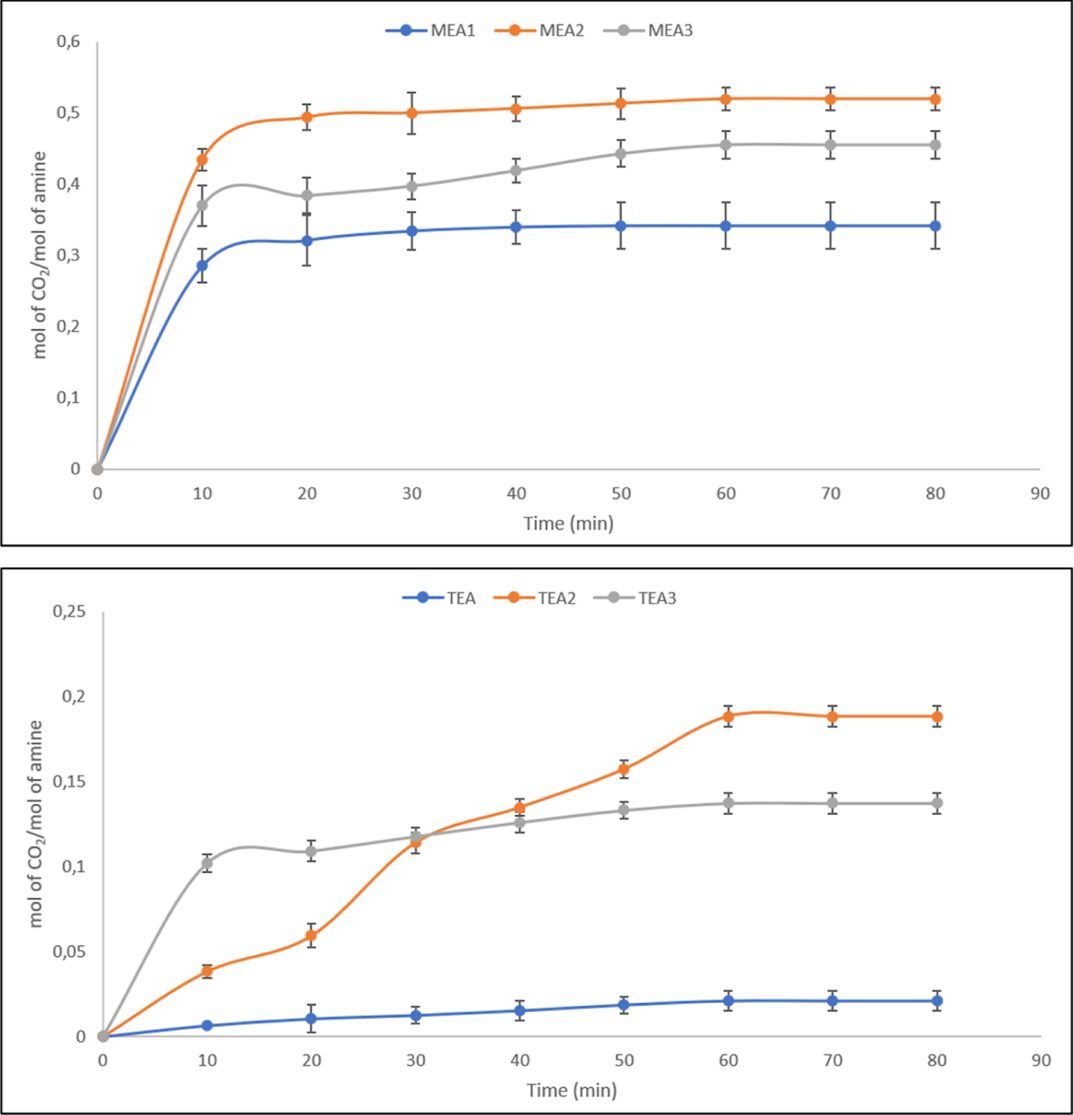
CO_2_ capture efficiency using system 1 (MEA (top) and TEA (bottom)). Error bars represent the standard deviation obtained from duplicate experiments.

An evaluation of the performance of amines in system 1 revealed that increasing the water content in the solution used for CO_2_ capture did not yield a beneficial effect. MEA and TEA exhibited the highest capture efficiency at a 1:1 ratio (MEA2 and TEA2). However, as the dilution ratio increased, a corresponding decrease in the efficiency was observed.

Analyzing the two amines individually, MEA exhibited a maximum capture efficiency of 0.519, slightly above the expected stoichiometric value of 0.50,^38^ within experimental uncertainty, suggesting a possible contribution from the reaction between water and CO_2_ producing bicarbonate (eq 6), and aligning with previously reported maximum capture results.^39,40^ However, this efficiency was attained only after 60 min, which can be considered a prolonged reaction time for a simple system. Similarly, TEA reached its highest capture efficiency within the investigated time window (60 min). However, the observed value (0.188) remained significantly lower than the theoretical stoichiometric benchmark than values reported in the literature for CO_2_ capture using TEA.^15,19^ This apparent plateau is assigned to kinetic limitations associated with gas–liquid mass transfer and the intrinsically slower reaction of TEA with CO_2_. Therefore, although no further increase was verified within the experimental duration, CO_2_ uptake in the jet system is expected to continue increasing over longer operation times as the system gradually approaches thermodynamic equilibrium.

In system 1, upon CO_2_ injection into the solution, the generated bubbles inside the solvent were heterogeneous in size and relatively large. The lack of control over bubble size may also be responsible for the extended time required to reach maximum efficiency and the low CO_2_ capture efficiency observed for TEA. Large bubbles do not provide an extensive gas–liquid interface, increasing mass transfer resistance and consequently hindering the absorption of CO_2_.^39^ For this reason, system 2, which uses a controlled system of microbubbles, was tested for CO_2_ capture by MEA and TEA.

Figure 5 exhibits the efficiency of CO_2_ capture for MEA and TEA at different amine concentrations for system 2.

**Figure 5:**
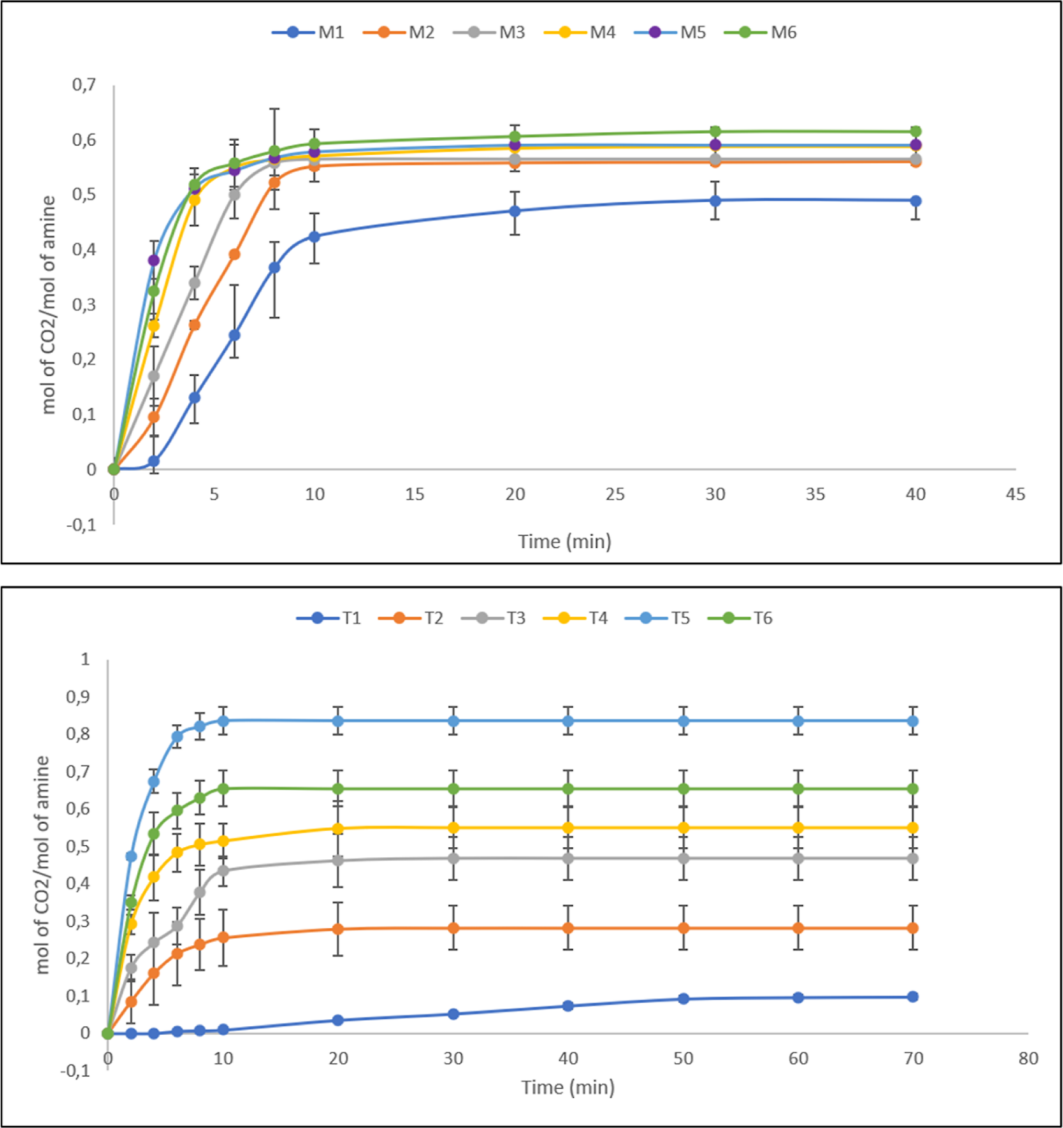
CO_2_ capture efficiency using system 2 (MEA (top) and TEA (bottom)). Error bars represent the standard deviation obtained from duplicate experiments.

Regarding the performance of MEA, the CO_2_ capture efficiency increased as the MEA concentration decreased, indicating that for system 2, the presence of water possessed a positive effect on CO_2_ capture. This phenomenon can be attributed to the fact that high MEA concentrations may increase both the density of MEA molecules and solution viscosity. Although microbubble injection can shorten the diffusion path by increasing the interfacial area, the increased viscosity at high MEA concentrations leads to a higher effective diffusion resistance.^38^ This finding is consistent with previous studies, which also demonstrated that higher MEA concentrations resulted in lower capture efficiencies. Yu and Chuang^41^ observed that solutions with lower amine concentrations exhibited superior efficiency. Specifically, a system without water achieved an efficiency of 0.20, whereas a system with the lowest amine concentration reached an efficiency of 0.54. Similarly, Tian et al.^38^ reported the same trend, noting that a 5 M MEA aqueous solution achieved a capture efficiency of 0.45 mol of CO_2_/mol MEA, while a 0.5 M MEA aqueous solution exhibited a higher efficiency of 0.48.

As previously discussed, based on the stoichiometry of CO_2_ capture by MEA via carbamate formation (eqs 1–3), the stoichiometric benchmark for CO_2_ uptake is 0.50 mol of CO_2_/mol of MEA. Except for the nonaqueous MEA solution, all tested systems exhibited final capture efficiencies slightly higher than this reference value. This behavior can be assigned to the presence of water, which improves CO_2_ uptake through hydration reactions leading to bicarbonate formation (eq 4). In this system, water therefore acts not only as a solvent but also as a functional component that promotes bicarbonate formation. Although this contribution is limited, it can slightly increase the overall CO_2_ capture capacity. Barzagli et al.^12^ also reported that the presence of water improves CO_2_ capture efficiency in MEA-based systems. In nonaqueous solutions, where CO_2_ capture occurs exclusively via carbamate formation, the measured efficiencies remained close to the stoichiometric benchmark. On the other hand, in aqueous solutions, where bicarbonate formation can also occur, the capture efficiencies were observed to be marginally higher. This limited contribution of bicarbonate formation is consistent with its relatively slow kinetics compared to the more kinetically favorable carbamate formation pathway.

The CO_2_ capture efficiencies obtained in the present study were higher than most values reported in the literature. In addition to the aforementioned studies that demonstrated lower efficiencies, de Ávila et al.^42^ reported a maximum efficiency of approximately 0.37, Yuan and Rochelle^35^ achieved an efficiency of 0.50, and Ge et al.^43^ obtained an efficiency of 0.47. All these studies employed CO_2_ capture systems different from the one utilized in the present work. By implementing our newly developed system, the highest dilution ratio (1:5) resulted in an efficiency of 0.615. This superior performance can be ascribed to the microbubble system, which facilitated CO_2_ delivery into the reaction system. Unlikely of system 1, the use of CO_2_ microbubbles allowed for a uniform dispersion within the amine solution, thus increasing the contact time between CO_2_ and the solvent and enhancing overall capture efficiency. The microbubble size also played a critical role, as smaller microbubbles provide a larger specific surface area, further improving CO_2_ absorption. In processes employing microbubble technology, optimizing both bubble formation and size is essential to maximize gas absorption efficiency.^44^ Another key advantage of this newly developed system is that the capture reactions occurred under ambient pressure and temperature conditions, which may contribute to reducing the overall process costs.

An important aspect to highlight concerns the reaction time, specifically, the duration required to attain the maximum CO_2_ capture efficiency. In the case of MEA using the newly developed system, the maximum efficiency was consistently achieved within 10 min across all conditions. This reaction time is significantly shorter than those typically reported in the literature for CO_2_ capture using the MEA. For example, Guo et al.^45^ observed the maximum efficiency after 60 min, while the system employed by Yang et al.^46^ enabled maximum efficiency to be reached in 30 min. Zhang et al.^47^ reported a substantially longer reaction time of approximately 400 min to attain peak efficiency. These findings underscore a key advantage of the system proposed in the present study as a shorter reaction time translates into enhanced process productivity and reduced operational costs.

With respect to the results of CO_2_ efficiency capture by TEA, as expected, the nonaqueous solution did not present significant CO_2_ capture. Only after 50 min did the efficiency possess a value close to 0.1. As previously mentioned, a TA does not have hydrogen atoms bonded to the nitrogen atom and TAs react with CO_2_ via a base-catalyzed hydration reaction. In other words, without water in the reaction medium in the presence of TAs, practically there is no CO_2_ capture.

The small uptake of CO_2_ from pure TEA may be due to physical absorption after a longer reaction time.^12,48^

The efficiency value increased nearly 3-fold (from 0.098 to 0.28) with the smallest amount of water utilized, confirming the critical role of water in CO_2_ capture. The efficiency continued to increase as the water content increased, reaching its peak at a dilution ratio of 1:4. This finding contradicts the trends reported in the literature. A decrease in the TEA concentration within the solvent was expected to reduce the number of TEA molecules available as active sites per unit volume, thereby limiting the chemical absorption of CO_2_.^49,50^ However, the observed opposite effect further highlights the efficiency of the novel microbubble system developed in this study. When the dilution was further increased to 1:5, the efficiency decreased from 0.83 to 0.66. In this case, this reduction in efficiency can be attributed to the lower concentration of TEA in the solution. These findings indicate that a dilution ratio of 1:4 represents the optimal limit for achieving a high CO_2_ capture efficiency for the system using TEA.

The theoretical CO_2_ capture efficiency for TEA, based on the equations governing the process (eq 4), is 1.0. The maximum efficiency achieved in the present study was 0.83. Despite being below the theoretical limit, this value surpasses most of the efficiency values reported in the literature for TAs, further demonstrating the superior performance of the novel system employed in this work. For instance, Gao et al.^51^ reported a TEA capture efficiency of 0.197, while the system utilized by Lu et al.^52^ achieved an efficiency of 0.442 for a TA. Similarly, Ge et al.^43^ obtained an efficiency of 0.634.

Similar to MEA, the TEA solutions achieved the highest CO_2_ capture efficiency within a short time frame, 20 min for lower dilutions and 10 min for higher dilution, confirming the advantage of the microbubble system in facilitating CO_2_ absorption. This system enhanced mass transfer efficiency, mitigating the mass transfer limitations between the gas and liquid phases that could otherwise lead to a great absorption rate only over long times.^45^

The analysis of Figure 4 also revealed that in both cases, the increase in efficiency became progressively smaller as it approached its maximum value. This phenomenon can be attributed to the increase in viscosity within the reaction medium. Although viscosity was not directly measured, visual observations indicated a noticeable increase in viscosity with decreasing water content, particularly when comparing nonaqueous and water-lean systems. Elevated viscosity leads to greater gas diffusion resistance, thereby reducing the CO_2_ absorption rate.^38,53^ At higher water contents, this negative effect was less pronounced, indicating that water also contributes to reducing solution viscosity, thereby facilitating mass transfer and enabling efficient CO_2_ absorption at ambient temperature and pressure. The lower increase in the level of CO_2_ capture toward the end of the reaction can also be attributed to the reduction in available active sites (amino groups) for CO_2_ absorption. A decrease in active sites results in fewer effective collisions between CO_2_ molecules and absorbents, consequently reducing the absorption rate.^54^

When directly comparing the performance of MEA and TEA, TEA achieved a higher absolute CO_2_ capture efficiency. However, taking the stoichiometry efficiency values into account, MEA exceeded its expected efficiency and was thus identified as the amine with the best overall performance. Other studies employing different capture systems have also reported superior results for MEA compared to other amines. Dubois and Thomas^55^ observed significant differences in absorption efficiencies among various amine solutions: very low for a TA, improved for a sterically hindered amine, and excellent for a primary (MEA). Similarly, Ge et al.^43^ found that the single-amine system using MEA exhibited a higher CO_2_ absorption efficiency (94.6%) compared to secondary (75.8%) and TAs (63.4%). The different behaviors observed for MEA and TEA can be further understood by considering their distinct CO_2_ reaction mechanisms and the stability of the corresponding intermediates. MEA, as a primary amine, reacts with CO_2_ through a zwitterion intermediate followed by carbamate formation, a pathway that involves two amine molecules and is kinetically favored owing to the relative stability of the carbamate species. As a result, CO_2_ absorption by MEA is less sensitive to gas–liquid mass transfer limitations.^56^ On the other hand, TEA, a TA, does not produce carbamate and instead acts as a base catalyst in the hydration reaction of CO_2_, leading to bicarbonate formation. This pathway involves lesser stable intermediates and slower intrinsic reaction kinetics, making the overall absorption process more dependent on CO_2_ availability at the gas–liquid interface.^56,57^ As a result, CO_2_ is absorbed more rapidly and in higher quantities in solutions containing the primary amine as MEA.^12,58^

Although TEA exhibited lower performance compared with MEA, its CO_2_ capture efficiency remains significant, as it is relatively close to the theoretical value and exceeds most values reported in the literature. Despite achieving the highest efficiency, MEA is characterized by high volatility and lower stability, which may hinder the capture process.^42^ In contrast, TEA, because of its molecular structure, demonstrates better stability. Furthermore, as a TA, TEA exhibits lower corrosiveness, reduced vapor pressure, and lower heat of absorption.^52^ These characteristics, combined with its relatively high absorption efficiency, position TEA as a potential alternative to MEA for CO_2_ capture applications using the new system.

### 3.23.2 Infrared Spectroscopy

The spectra of the amines prior to and after CO_2_ absorption are presented in Figure 6. MEA exhibits characteristic vibrational modes at 3348 and 3282 cm^–1^ related to N–H stretching, at 2914 and 2854 cm^–1^ (symmetric stretching of CH_2_), at 1597 cm^–1^ (N–H rocking), at 1353 cm^–1^ corresponding to NH_2_ twisting, at 1074 cm^–1^ (C–N stretching), at 1024 cm^–1^ (C–O stretching) as well as at 933 cm^–1^ (C–N–H out-of-plane wagging and C–NH_2_ twisting).^59,60^ Relation to TEA, characteristic peaks at approximately 3300 cm^–1^ corresponding to N–H stretching, at 2945, 2877, and 2812 cm^–1^ (symmetric stretching of CH_2_), at 1400 and 1149 cm^–1^ (asymmetric stretching of C–N), at 1355 and 1278 cm^–1^ (CH_2_ bending), and at 1025 cm^–1^ attributed to the CH_2_ rocking were observed.^61^

**Figure 6:**
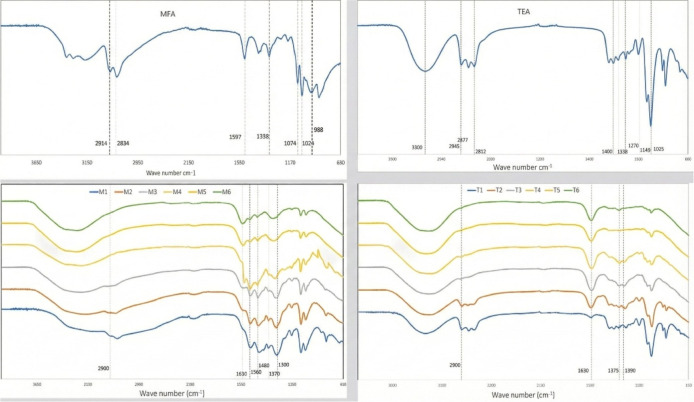
FTIR spectra of pure amines (top) and of the solutions after CO_2_ capture process (bottom).

Most of the IR measurements were conducted for aqueous solution. The O–H stretching vibrations of H_2_O exhibit strong absorption in the range of 3200–3700 cm^–1^. Due to hydrogen-bonding interactions, these absorption peaks are notably broad. Additionally, the N–H, C–H, and O–H stretching vibrations of aminoalcohol molecules also contribute to absorption within this spectral region, commonly referred to as the “hydrogen stretching region”, and distinguishing between these individual contributions is challenging, as the water absorption peak predominates. For pure TEA, it was possible to note that this peak was more prominent. Consequently, the wavenumber range of a 3000–4000 cm^–1^ region is not particularly informative for TEA, as the strong and broad O–H stretching band of water masks the characteristic vibrations of the amine. In the case of pure MEA, this peak was not so strong. Therefore, it is possible to state that this peak in the MEA samples after the absorption process is owing to water.^59^

In both cases, the adsorption of CO_2_ onto amines led to the formation of NH_3_^+^/NH_2_^+^ species, whose stretching vibrations can be visualized around 2900 cm^–1^ and bending vibrations at 1630 cm^–1^. Specifically for MEA, the principal findings after CO_2_ capture were band asymmetric and symmetric stretching vibrations of the COO^–^ group in carbamates at 1560 cm^–1^ and 1480 cm^–1^.^62,63^ Additionally, the MEA spectra after the absorption process confirmed the formation of bicarbonate, since peaks at 1375 cm^–1^ and 1300 cm^–1^, which can be ascribed to the stretching vibration of CO and bending vibration of COH of the HCO_3_^–^ molecule, were also noted.^64^ For TEA, as there is no formation of carbamate during the CO_2_ absorption process, only the corresponding peaks for bicarbonate (1350 cm^–1^ and 1280 cm^–1^) were verified as new peaks after the capture process.

### 3.3 Modeling Results

The mathematical model developed in this study was tested against experimental data from CO_2_ capture experiments using different dilutions of MEA and TEA in water. The best-fitting results were obtained by minimizing the sum of the squared errors between the experimental data and model predictions. The modeling results are presented in Figure 7.

**Figure 7:**
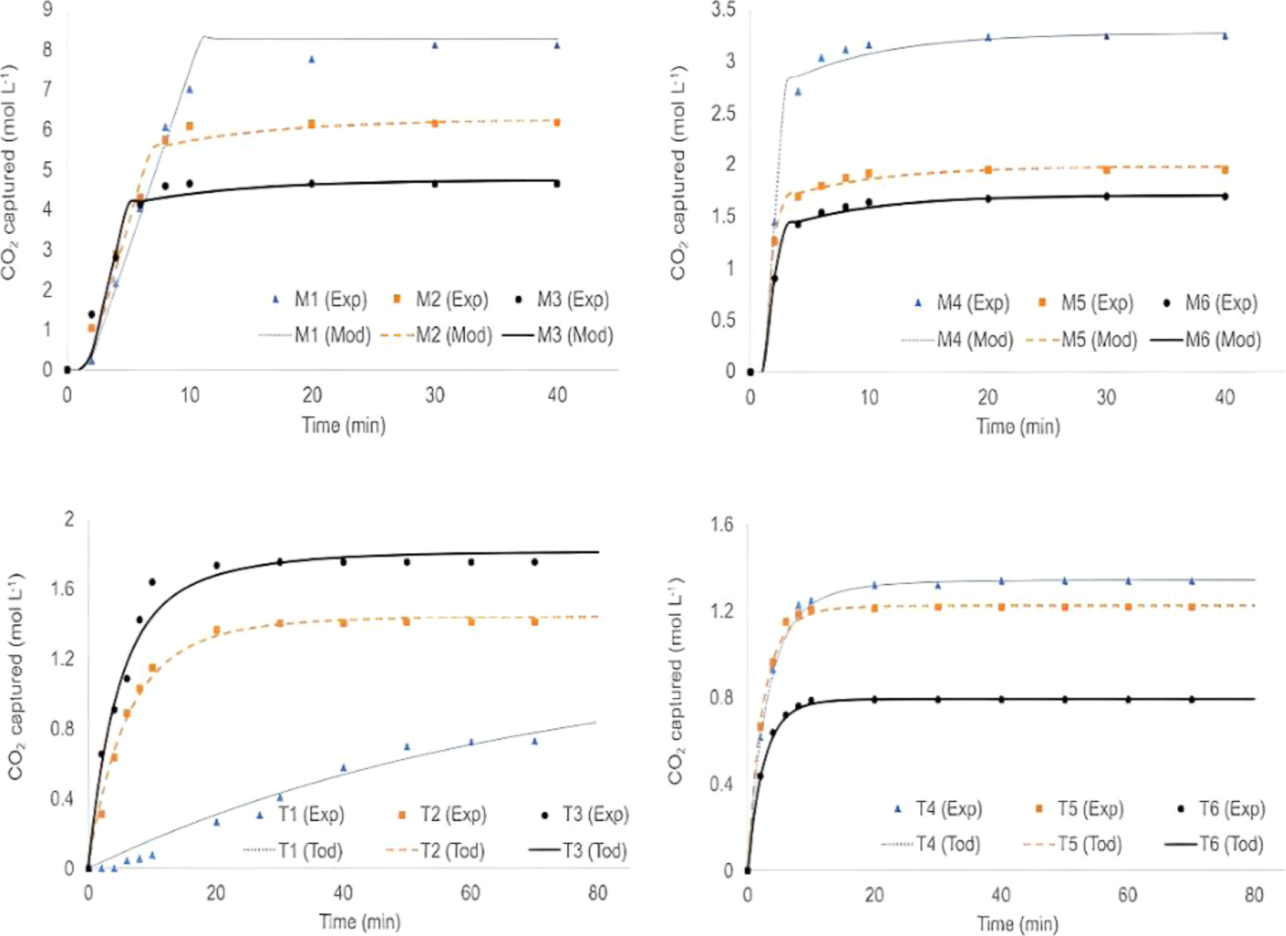
Modeling results for CO_2_ capture using MEA (top) and TEA (bottom).

A good agreement between the model and experimental data can be observed in Figure 7, with average *R* ^2^ values of 0.986 and 0.989 for the MEA and TEA studies, respectively. Table 3 shows the kinetic parameters that were obtained.

**Table 3:** Kinetic Parameters for CO_2_ Capture by MEA and TEA

parameter	value	unit
MEA		
<*k*_1f_	3.04 × 10^4^	L^2^ mol^–2^ s^–1^
*k*_1r_	0.00	L mol^–1^ s^–1^
*k*_2f_	3.83 × 10^–4^ – 1.76 × 10^–3^	L mol^–1^ s^–1^
*k*_2r_	1.71 × 10^–3^ – 2.17 × 10^–3^	s–1
TEA		
*k*_3f_	1.07–3.54	L mol^–1^ s^–1^
*k*_3r_	1.00 × 10^7^	L mol^–1^ s^–1^
*k*_4f_	1.03	L mol^–1^ s^–1^
*k*_4r_	5.71 × 10^–4^ – 1.99 × 10^–3^	s^–1^

The kinetic data obtained in this study are consistent with values reported in the literature.^34,35^ These findings indicate that the newly developed microbubble apparatus is well designed, that the measured kinetic data are reliable, and that the absorption rate/kinetics model employed in this work is both accurate and efficient.

Some fluctuations are observed in the parameters *k*_2f_, *k*_2r_, *k*_3f,_ and *k*_4r_ within the ranges listed in Table 2. These fluctuations vary from one dilution to another, indicating that the amine/water ratio influences the reaction rates. The parameter *k*_1r_ = 0 suggests the irreversibility of the reaction between CO_2_ and MEA (CO_2_ + 2MEA → MEACOO^–^ + MEAH^+^) under the studied conditions. The high value of *k*_3r_ compared to *k*_3f_ indicates the predominance of the reversible step in this reaction, which is expected since it involves two ions (TEAH^+^ and OH^–^). Similarly, the ions MEACOO^–^, AH^+^, and HCO^–^ are also expected to react rapidly in their reversible steps due to their high reactivity; however, this was not confirmed by the obtained rate constant.

Values even so, it is important to note that ions such as OH^–^ and HCO_3_^–^ can be readily consumed in parallel or sequential reactions whose rates may compete with those considered in this study. To reduce the number of parameters and enhance the robustness of the model, the reaction mechanisms were simplified into two reactions each, which proved to be sufficient for achieving a good-quality fit. The fitted values of the mass transfer coefficient are related in Table 4.

**Table 4:** Mass Transfer Parameters^a^

parameter	value in L s–1					
*k*_TM_	9.14 × 10^–3^	1.07 × 10^–2^	1.24 × 10^–2^	4.24 × 10^–2^	3.86 × 10^–2^	2.64 × 10^–2^
*k*_TT_	6.10 × 10^–6^	7.99 × 10^–5^	1.02 × 10^–4^	1.46 × 10^–4^	1.94 × 10^–4^	1.75 × 10^–4^

a ^[^CO_2_^]s^ = 0.75–1.47 mol L^–1^ (solubility in TEA).

A consistent trend was verified for both *k*_TM_(MEA) and *k*_TT_(TEA), with low values in pure MEA/TEA, followed by an increase upon dilution and a slight decrease at higher water contents. This behavior is consistent with competing effects reported in the literature rather than direct experimental evidence obtained in the present study.

On the one hand, dilution diminishes solution viscosity, which is expected to improve mass transfer. On the other hand, increased water content may affect bubble stability and effective interfacial area, potentially decreasing mass transfer efficiency.^35^ Although physical properties such as viscosity were not directly measured, their influence is discussed qualitatively based on literature trends, and the improved mass transfer behavior inferred in this study is supported by the significantly shorter absorption times and the strong agreement between experimental results and model predictions.

A similar qualitative trend was noted for the fitted CO_2_ solubility values in TEA, which increased with dilution and decreased at higher water contents, remaining within the ranges reported in Table 4.

### 3.4 Thermodynamic Properties

The conductor-like screening model (COSMO), originally proposed by Klamt, represents an efficient implementation of dielectric continuum solvation methods within quantum chemical calculations. Its extension, the COSMO for Real Solvents (COSMO-RS), enables the prediction of thermodynamic equilibria in fluid systems by integrating quantum chemical data with a statistical thermodynamics framework. In opposite to traditional group contribution methods, COSMO-RS offers the distinct advantage of being an a priori predictive model that does not rely on experimental input, allowing for extrapolation to novel chemical systems with qualitative accuracy. Furthermore, COSMO-RS presents the ability to discriminate among distinct isomers, improving its applicability. As a result, the model has gained widespread adoption within the chemical engineering community.^65,66^ In COSMO-RS, the screening (polarization) charge densities of molecular surface segments are calculated using quantum chemical methods within a virtual conductor environment. These charge distributions, represented as σ-profiles, characterize the molecule’s surface polarity and encapsulate essential chemical information required to predict electrostatic, hydrogen-bonding, and dispersion interactions in fluid systems.^67^

In the COSMO-RS simulation study of the present work, aqueous MEA and triethanolamine (TEA) were evaluated as solvents for CO_2_ absorption in the microbubble system. This section used COSMO-RS to predict the solubility of CO_2_ in the solvent components at a temperature of 298.15 K according to the predicted interactions among amine, CO_2_, and water, as the interactions between solvents and CO_2_ significantly contribute to the solubility of CO_2_. Such a parameter is widely recognized as a standard in the CO_2_ absorption technology owing to its relevance for assessing capture efficiency. Therefore, the sigma profiles were utilized to comprehend the electrostatic interactions among water, MEA, TEA, and CO_2_. The sigma profile (Figure 8 for the water + MEA + CO_2_ system and Figure 9 for the water + TEA + CO_2_ system) elucidates the CO_2_ solubility trend in aqueous MEA and TEA.

**Figure 8:**
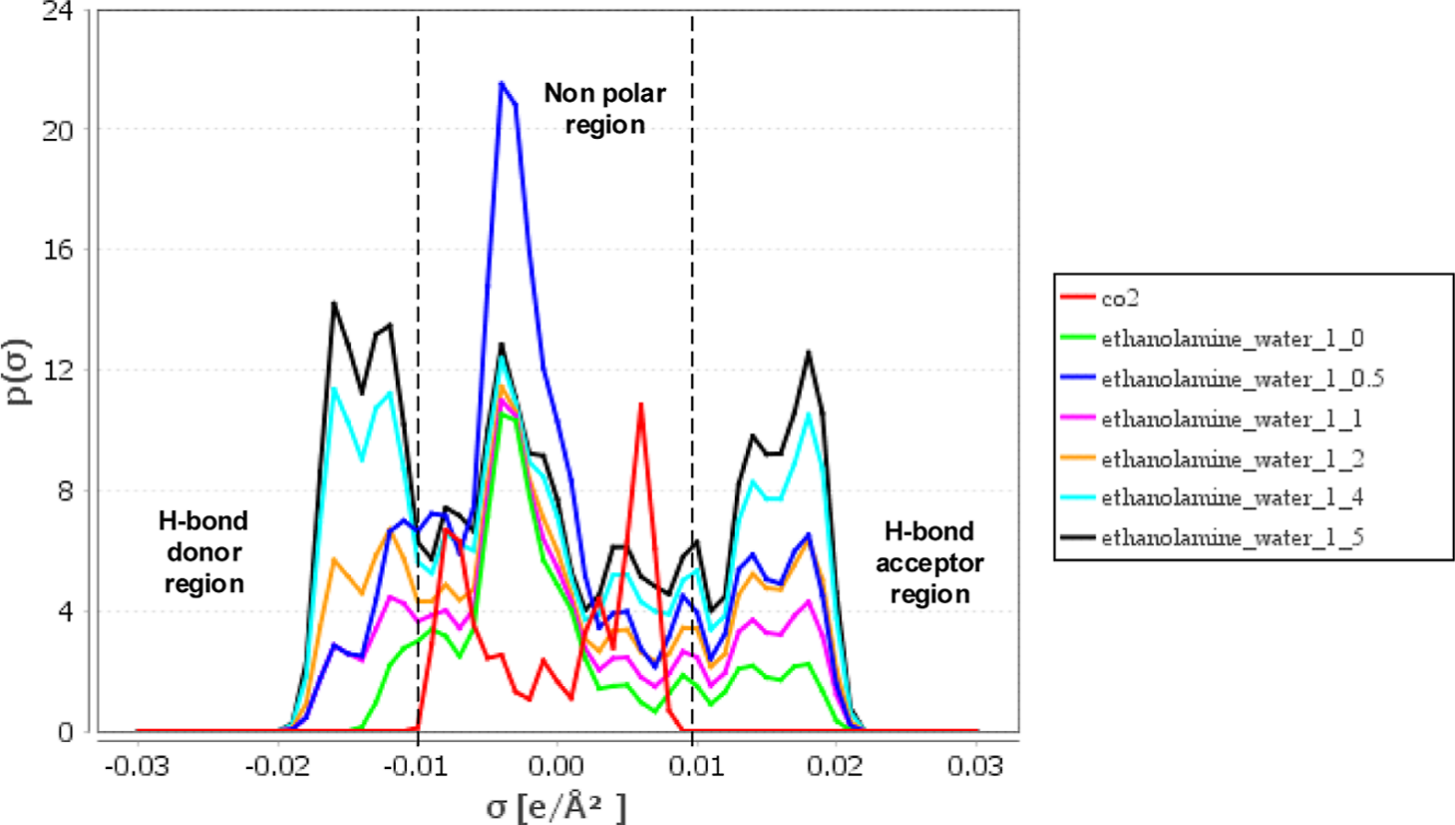
σ-Profiles for CO_2_ and aqueous MEA system.

**Figure 9:**
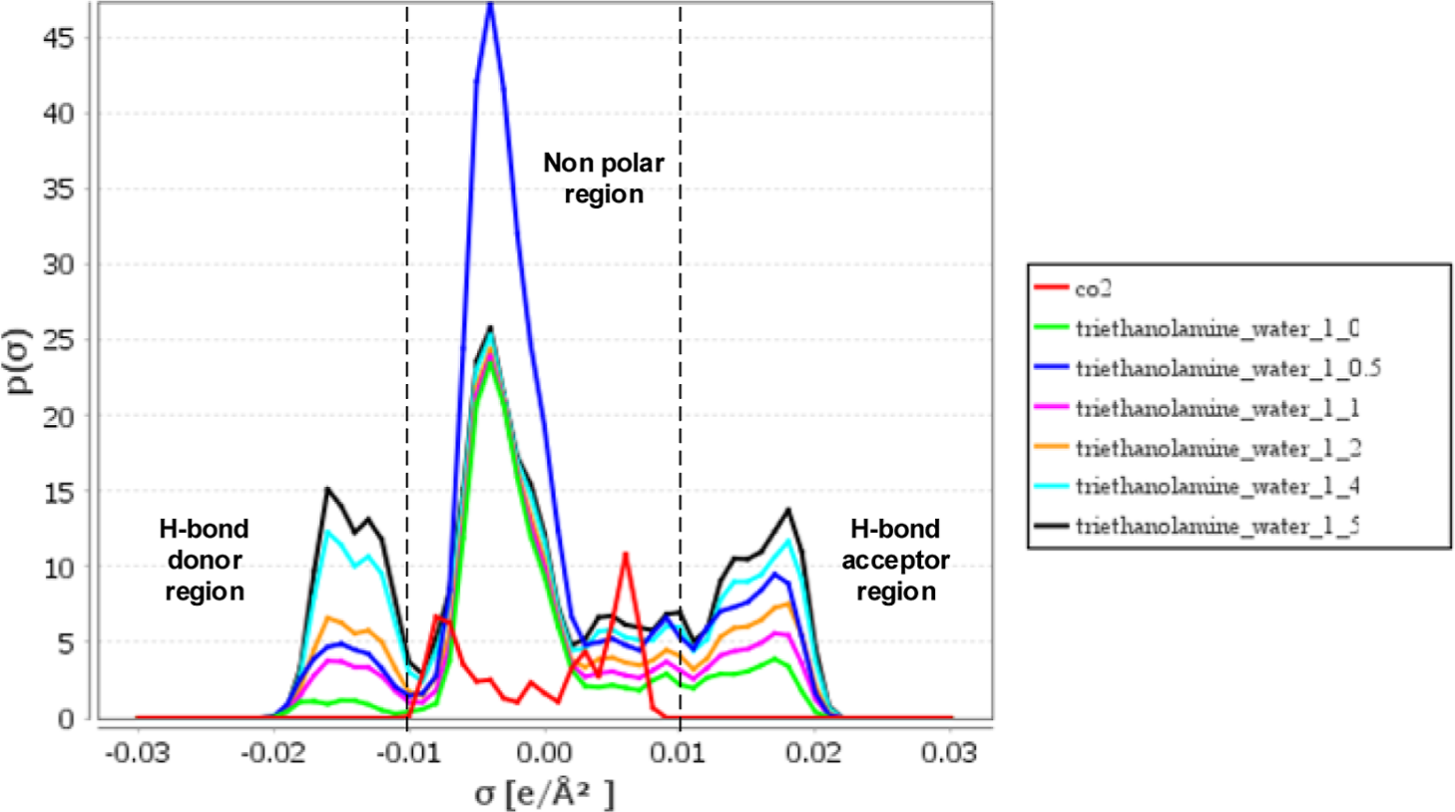
σ-Profiles for CO_2_ and aqueous TEA system.

It is important that each component of the solvent system is totally compatible and mutually soluble as this combination significantly influences critical properties such as viscosity, CO_2_ solubility, and heat capacity. This unique solvent system, composed of water, MEA, or TEA, facilitates synergistic interactions between them, thereby enhancing CO_2_ capture efficiency and improving the overall solvent performance.^68^

The COSMO-RS σ-profile histogram can be qualitatively segmented into three principal regions based on specific cutoff values: the HB donor region (σ < −0.0082 e/Å^2^), the hydrogen acceptor (HA) region (σ > +0.0082 e/Å^2^), and the nonpolar region, defined within the intermediate range (−0.0082 < σ < +0.0082 e/Å^2^).

The nature of the molecules is responsible for the variation of the position, width, and the height of the peaks.^69^ As can be visualized in Figures 8 and 9, CO_2_ is entirely in the nonpolar region enabling potential van der Waals interactions with the nonpolar regions of MEA and TEA. Moreover, CO_2_ presents good molecular symmetry as it possesses peaks in both positive and negative regions.^70^ However, the highest peak is in the positive region, which means that, theoretically, the compounds with a more negative potential at a certain σ will attract more CO_2_.^71^

Observing MEA σ-profiles, they present a pronounced positive polarization charge density at +0.014 and +0.018 e/Å^2^, assigned to the nitrogen atom of the molecule’s primary amine group indicating its capacity to function as a HB acceptor and reflecting its basic character. Besides, MEA exhibits a distinct peak around −0.005 e/Å^2^ within the nonpolar region, related to the alkyl groups bonded to the nitrogen atom. Furthermore, peaks at −0.013 and −0.016 e/Å^2^ can be noted, corresponding to the acidic hydrogen atom of the hydroxyl group, which characterizes the molecule’s primary alcohol functionality. The highest peaks are in the negative region, confirming the significant ability of aqueous MEA in absorbing CO_2_. The same pattern was reported by.^68,72^

The highest peaks of the HB and HA regions belong to the system MEA + water with the highest dilutions, reinforcing the importance of water in the process of CO_2_ capture. According to Mohd Rasdi et al.,^73^ the OH^–^ groups of water are located in the > +0.0086 e/Å^2^ region, functioning as HB acceptors capable of engaging in hydrogen bonding. Similarly, the H^+^ ions of water fall within the < −0.0086 e/Å^2^ region—corresponding to the HB donor domain—indicating their propensity to form hydrogen bonds with acceptor sites. In both cases, water cooperates with amine to increase the capacity of capture CO_2_. On the other hand, the lowest dilutions exhibited the highest peak in the nonpolar region, indicating that a lower quantity of water leads to a greater presence of van der Waals forces than hydrogen bonds.

Analyzing TEA σ-profiles, it is possible to verify a similar trend that was observed for MEA. The main difference is the intensity of the peaks in the nonpolar region. TEA presented higher peak intensity in this region for owning more alkyl groups bonded to the nitrogen atom than MEA. Hence, TEA has a higher tendency in bonding with CO_2_ by van der Waals forces than MEA. Regarding HB and HA regions, the intensities of the peaks are not quite different. TEA also showed a tendency of acting as a HB acceptor (+0.018 e/Å^2^) due to its basic character and a tendency of HB acceptor (−0.016 e/Å^2^), the acidic hydrogen atom of the hydroxyl groups. Water also exhibited an essential role when combined with TEA. The peaks of HB and HA increased with the increase of the water content in the system. The rise in peaks, mainly in the negative area, is directly linked to CO_2_’s solubility in the solvent since more hydrogen bonds will be formed, contributing to the van der Waals forces (nonpolar region) and increasing CO_2_’s solubility.

The sigma potential of aqueous MEA, TEA, and CO_2_ is presented in Figures 10 and 11, corresponding to the water + MEA + CO_2_ and water + TEA + CO_2_ systems, respectively. As a measure of the solvent’s response to molecular surface polarity, the sigma potential can also serve as a qualitative tool for assessing solvent suitability in CO_2_ absorption applications. The sigma potential profile of CO_2_ is symmetrical and exhibits a parabolic-like shape, and since the sigma potential of CO_2_ is positive across nearly the entire σ range under investigation, with an approximately zero potential between −0.01 and +0.01 e/Å^2^, compounds possessing more negative sigma potentials within specific σ intervals are expected to exhibit stronger attractive interactions with CO_2_.^74^ Such information confirms the high capacity of aqueous MEA and TEA in absorbing CO_2_, as both systems presented negative sigma potentials in HB and HA regions.

**Figure 10:**
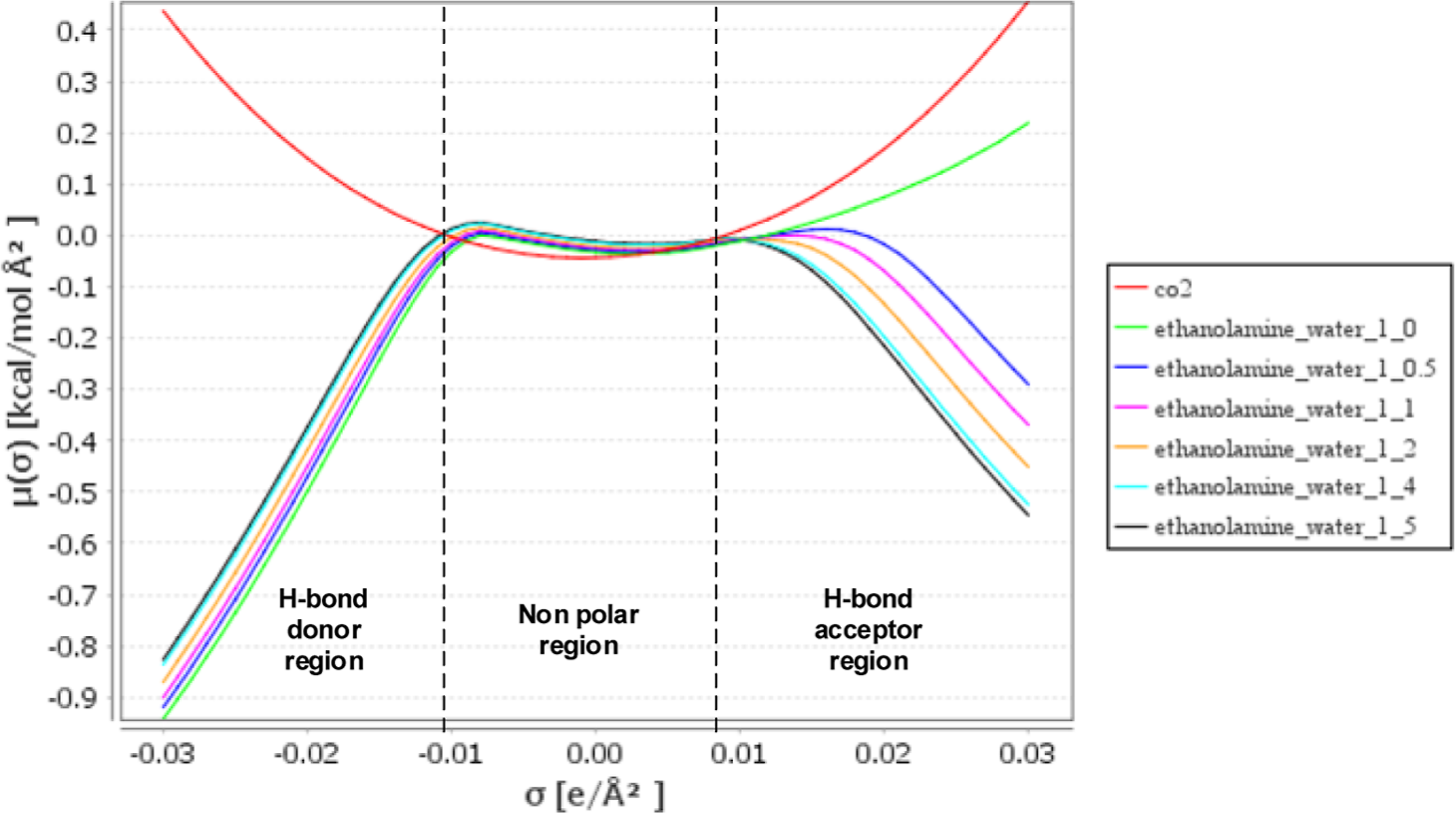
σ-Potentials for CO_2_ and aqueous MEA system.

**Figure 11:**
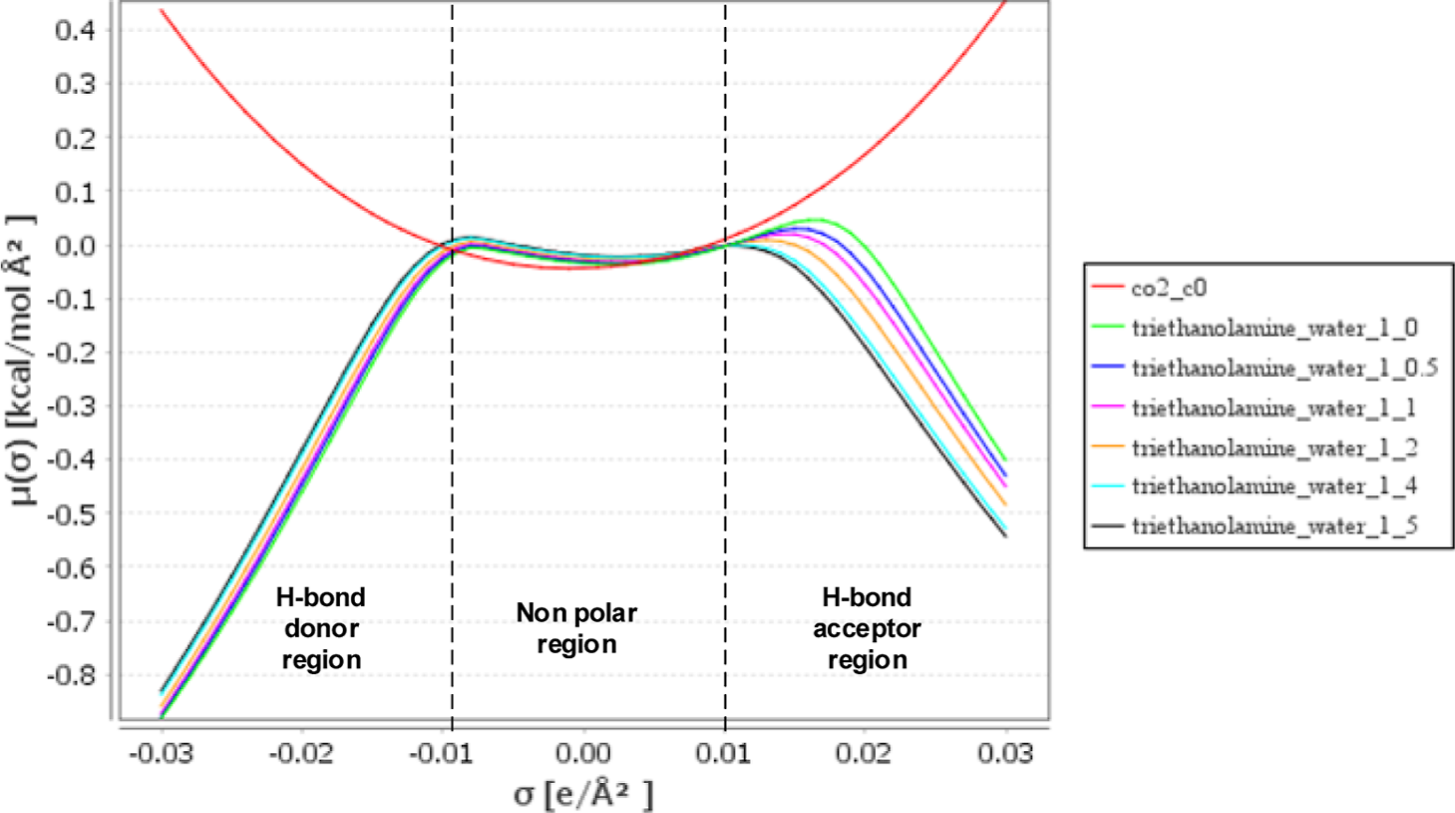
σ-Potentials for CO_2_ and aqueous TEA system.

The results achieved for σ-profiles and σ-potentials corroborated with the results of CO_2_ capture and modeling, reinforcing the efficiency of the microbubble system employed to capture CO_2_.

## 4 Conclusions

The microbubble-based CO_2_-injection configuration performed high absorption efficiencies under ambient conditions and in a short operational time, highlighting its potential for energy-efficient and scalable carbon capture applications. Using the microbubble system, the MEA-based solvent reached capture efficiencies approaching or slightly exceeding the stoichiometric theoretical limit, an unusual behavior reported in conventional capture systems, a behavior rarely reported for conventional capture systems. In contrast, TEA exhibited efficiencies close to its stoichiometric theoretical limit, which is not typically observed in the conventional systems. This enhancement observed for TEA is primarily attributed to improved mass transfer, compensating for its inherently slow reaction kinetics. Additionally, the highest efficiencies were attained under conditions of high dilution and short reaction times, indicating that the system enables reagent conservation and high productivity, which may contribute to the cost reduction in large-scale applications. Kinetic modeling and thermodynamic properties validated the experimental findings, consolidating the effectiveness of the proposed microbubble system.
